# Abdominal drain straying into right atrium: a case report

**DOI:** 10.1186/s40792-019-0685-7

**Published:** 2019-08-06

**Authors:** Junya Toyoda, Hitoshi Sekido, Kazuhisa Takeda, Tetsuya Shimizu, Goro Matsuda

**Affiliations:** 1 Department of Surgery, National Hospital Organization Yokohama Medical Center, 3-2-60, Harajuku, Totuka-Ku, Yokohama, Kanagawa 245-8575 Japan; 2 Department of Surgery, NTT Medical Center Tokyo, 5-9-22, Higashi-Gotanda, Shinagawa-Ku, Tokyo, 141-8625 Japan

**Keywords:** Hepatectomy, Abdominal drain, Right atrium, Straying, Fibulin thrombus

## Abstract

**Background:**

A drain exchange with the use of a guidewire may be accompanied by serious complications.

**Case presentation:**

This case involved an 86-year-old man with overlapping cancers of intrahepatic cholangiocarcinoma and perihilar cholangiocarcinoma. A left hepatectomy, a left caudal lobectomy (with a medial hepatic vein preservation), an extrahepatic bile duct resection, and a right hepatojejunostomy were performed. The abdominal drain was placed into the hepatectomy side. Bile leakage occurred on the seventh day after the surgery, and the drain was exchanged. Since the bile leakage was still detectable via a computed tomography (CT) scan on the 15th postoperative day, the drain was exchanged again. On the next day, blood had discharged from the drain. A CT scan revealed that the tip of the drain was straying into the right atrium (RA) and the drain was removed from the inferior vena cava (IVC) under general anesthesia. One week later, a fiburin thrombus was observed from the IVC to the RA via the use of transthoracic echocardiography. A right atrial incision, a thrombus removal, and a middle hepatic vein merging section closure surgery were performed. Afterward, the patient’s general condition gradually improved, and he was transferred to the hospital for rehabilitation.

**Conclusion:**

More careful guidewire operations are necessary at the time of the exchange of the drain to prevent the drain from being placed too close to blood vessels.

## Background

When long-term drainage is required after a gastrointestinal cancer surgery, a drain exchange is regularly required to maintain the effective drainage [1]. At that time, it is important to place the tip of the drain in the proper position by using a guidewire, but there may be serious complications that are associated with the guidewire operation. Herein, we report a novel case in which the middle hepatic vein was punctured by the abdominal drain exchange, after which the tip of the drain strayed into the right atrium (RA).

## Case presentation

**Fig. 1:**
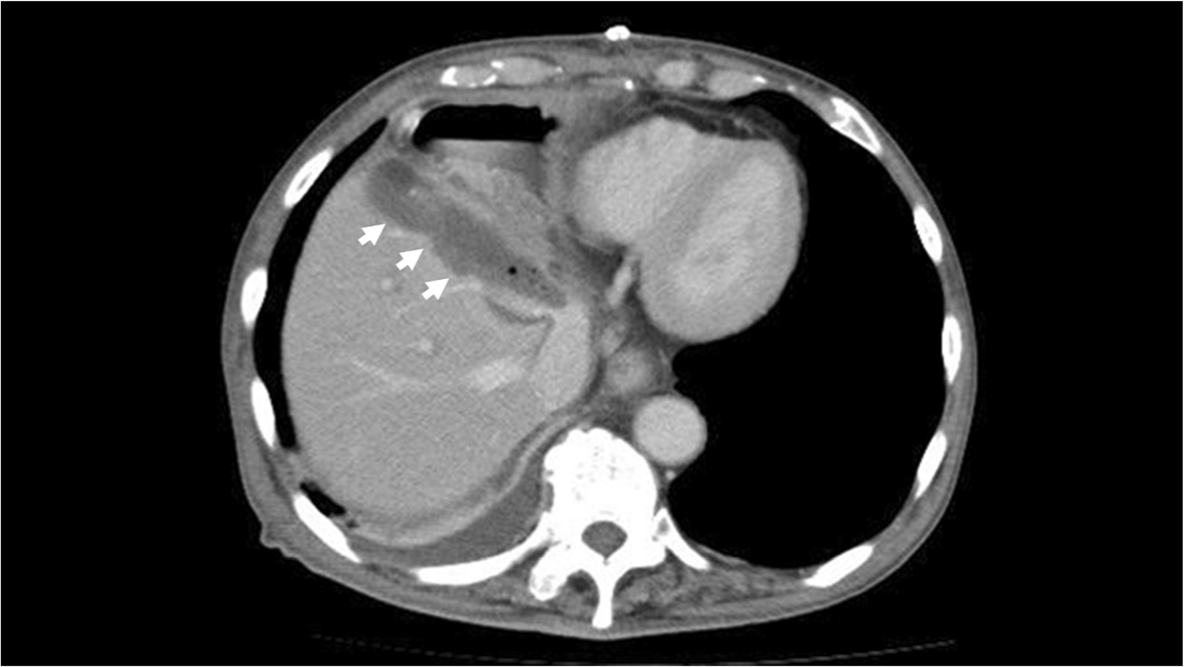
An enhanced abdominal CT scan showed fluid collection at the transected liver surface (arrow)

**Fig. 2:**
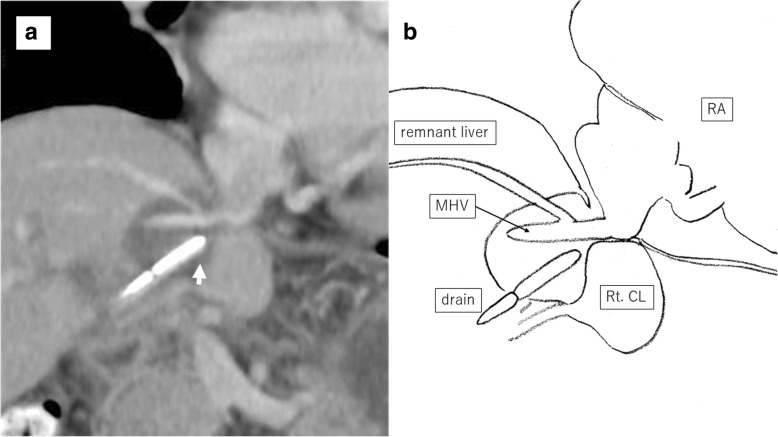
An enhanced abdominal CT scan before exchanging the drain showed the tip of the drain pointed to the bifurcation of the MHV (arrow, **a**). Schema (**b**). *MHV* middle hepatic vein, *Rt*. *CL* right caudate lobe, *RA* right atrium

**Fig. 3:**
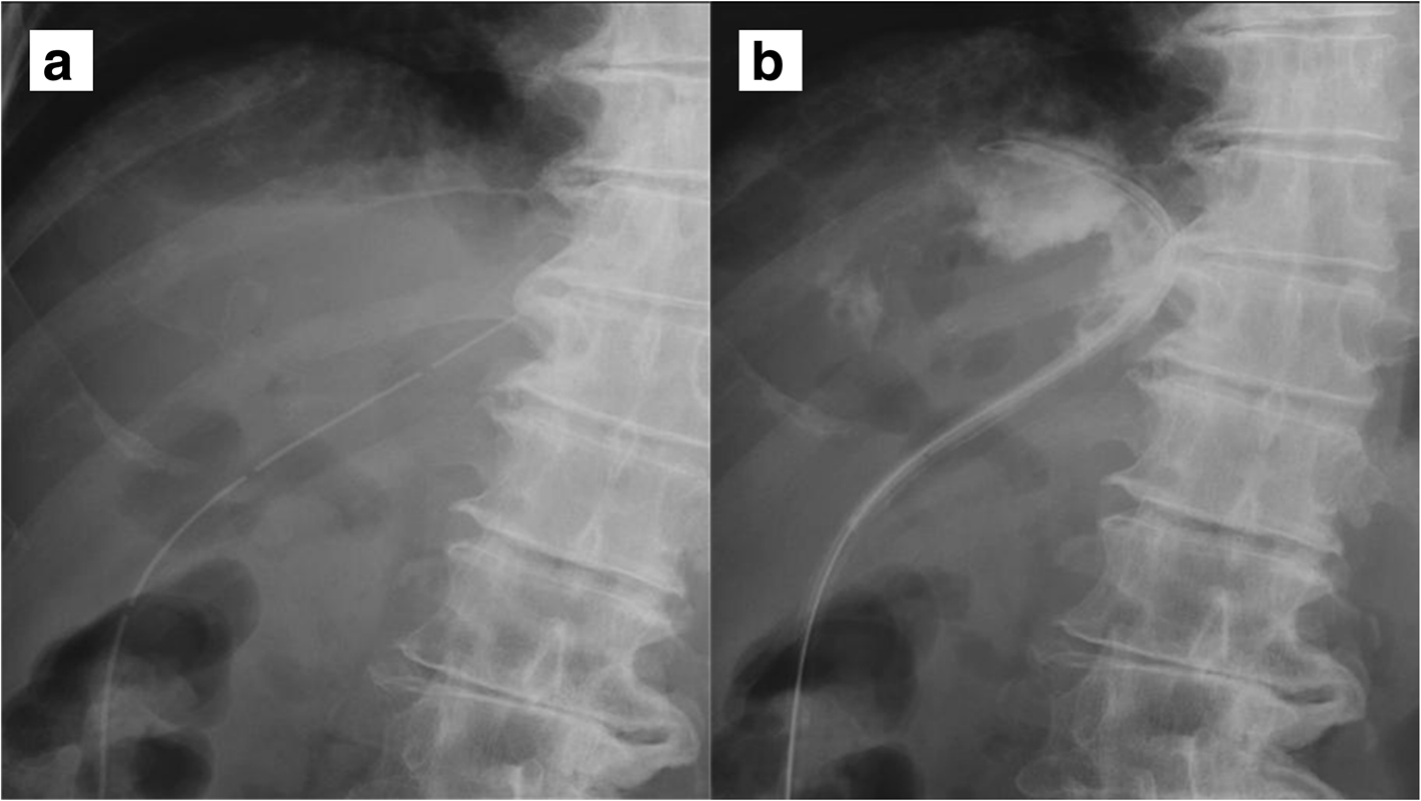
Fluoroscopy showed the drainage tube before exchange (**a**) and after (**b**). The drainage tube is thought to have strayed into the MHV after that. *MHV* middle hepatic vein

**Fig. 4:**
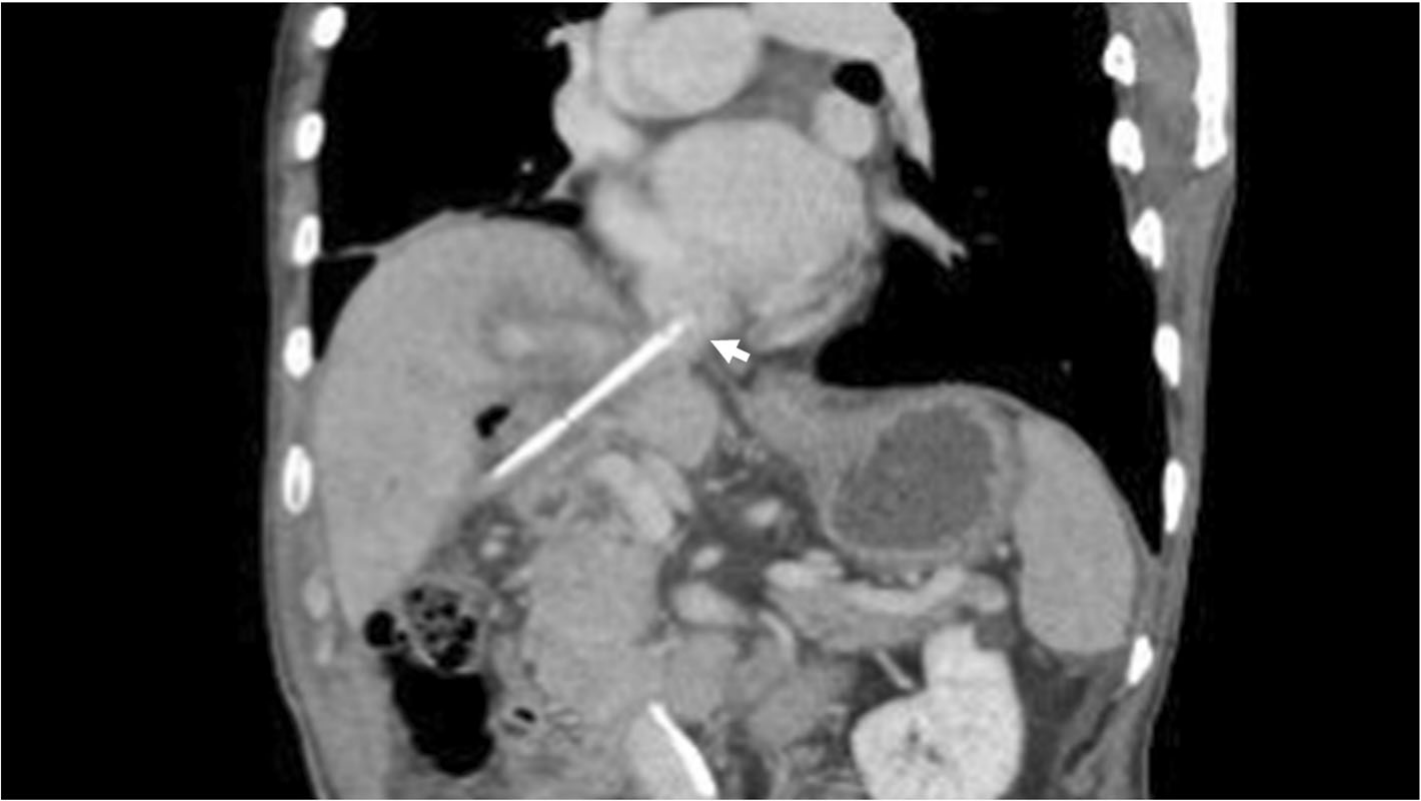
An enhanced abdominal CT scan showed the tip of the drain perforating the IVC and straying into the RA (arrow). *IVC* inferior vena cava, *RA* right atrium

**Fig. 5:**
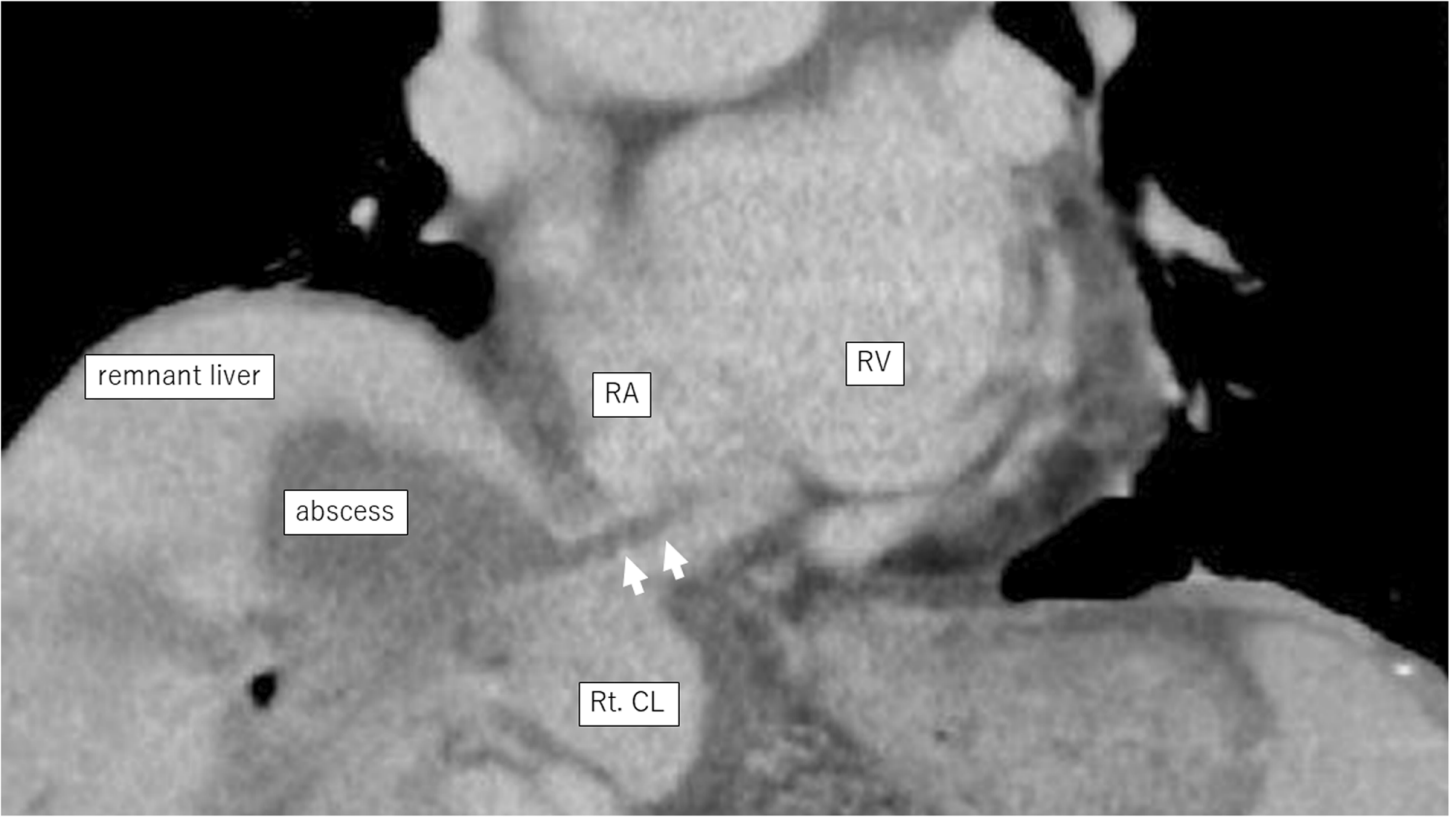
An enhanced abdominal CT scan showed the low density area continuous from the abscess to the RA (arrow). *RA* right atrium

**Fig. 6:**
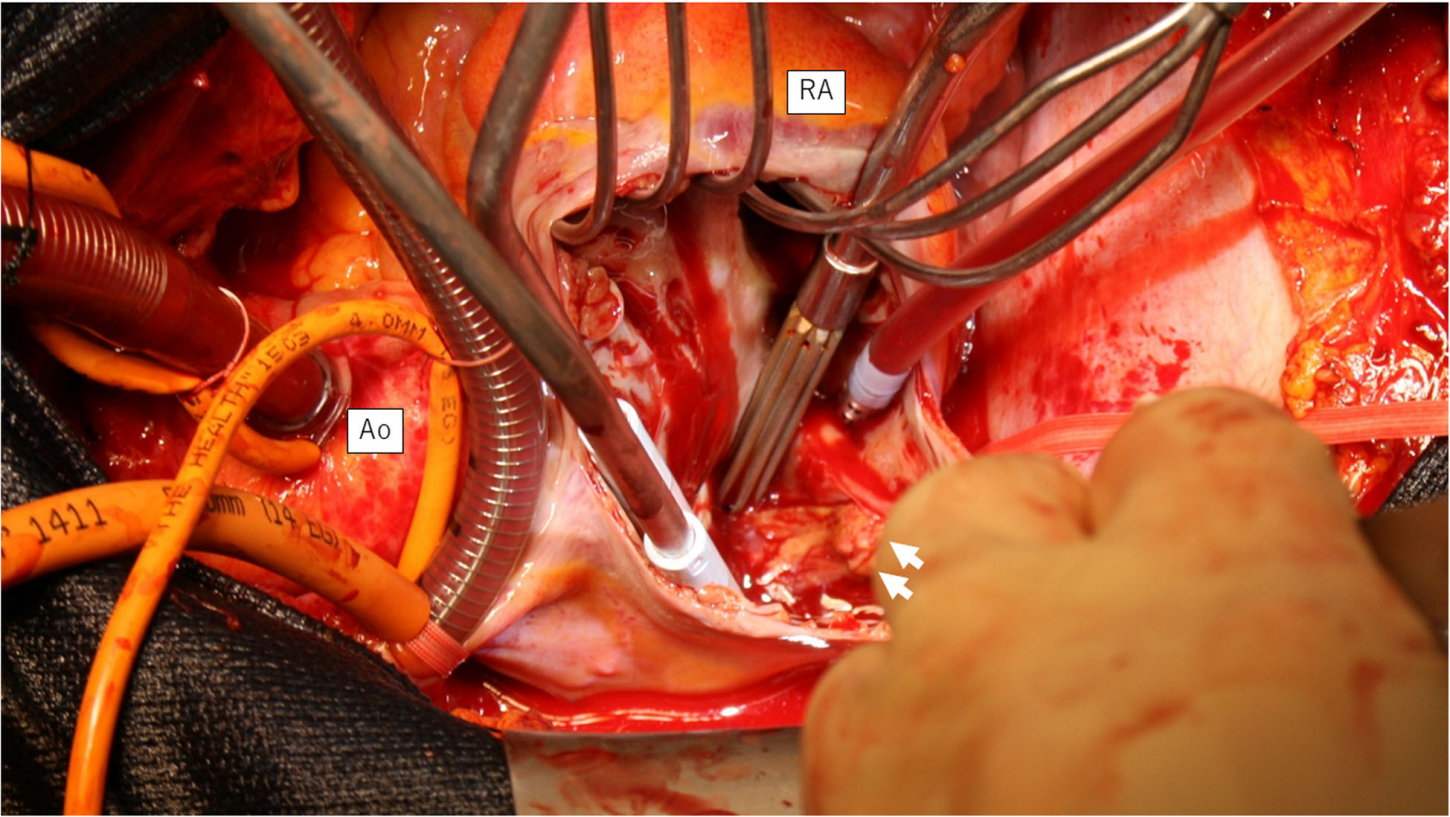
Intraoperative findings: the yellow-brown tumor originated from the IVC and had a size of 3 × 1 cm (arrow). *IVC* inferior vena cava

**Fig. 7:**
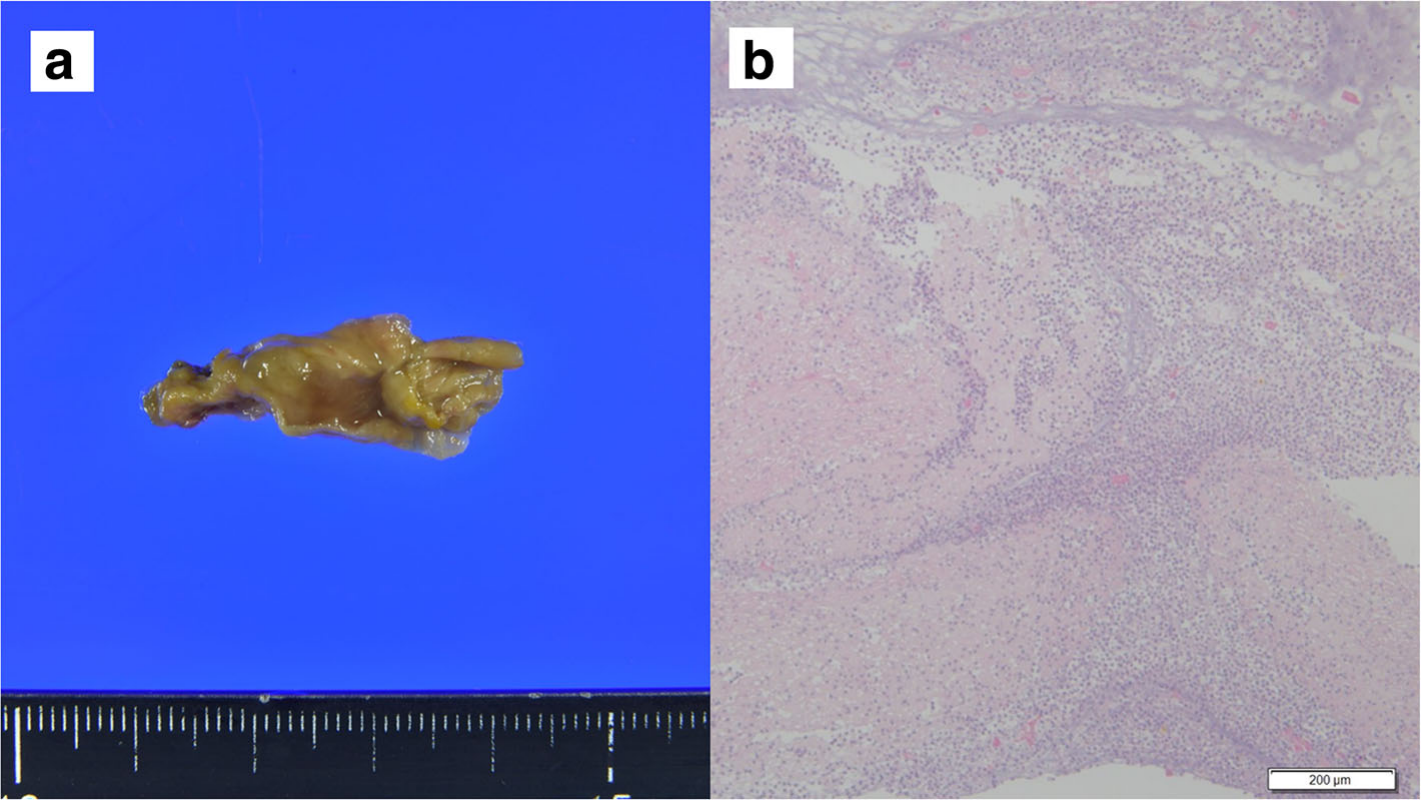
The tumor was yellow tone, measuring 3 × 1 × 0.7 cm (**a**). Histological findings HE × 40: most of the tissue was fibrin thrombus, and a collection of neutrophils was recognized mainly from the margin (**b**)

An 86-year-old man was observed to have skin erosion and high levels of bilirubin in the urine, after which he visited our hospital. Obstructive jaundice was referred and both of liver mass and biliary mass were diagnosed. He underwent a left hepatectomy, a left caudal lobectomy (with a medial hepatic vein preservation), an extrahepatic bile duct resection, and a right hepatic cholangiojejunostomy. During operation, the root of LHV and the branch of MHV were ligated twice. The 4Fr silicon tube (Atom Medical, Tokyo, Japan) was placed as internal biliary stent. The operation time was 349 min and the intraoperative bleeding was 720 ml. A 19 Fr closed, low-pressure, continuous suction silicone drain (Johnson & Johnson, New Jersey, USA) was placed on the transected liver surface. Histological diagnosis of liver mass was adenocarcinoma (moderately differentiated type, tub2), and that of biliary mass was papillary adenocarcinoma (pup) and adenocarcinoma (moderately differentiated type, tub2). Therefore, we diagnosed as double primary cancers of intrahepatic cholangiocarcinoma and perihilar cholangiocarcinoma. On the seventh postoperative day, a computed tomography (CT) scan showed the presence of fluid collection at the transected liver surface (Fig. 1). We then diagnosed the bile leakage caused by injury of peripheral bile duct and abscess under the right diaphragm had formed. The drain was exchanged for a 12 Fr silicone drain (Create Medic, Kanagawa, Japan) by using a 0.035 in angle-stiff type guidewire (TERUMO, Tokyo, JAPAN) under fluoroscopy. The bile leakage was under control by the drainage. Therefore, we did not consider biliary drainage with ENBD or ERBD via ERCP. However, a fever then occurred, due to the poor drainage of an abscess under the right diaphragm. The tip of the drain was reduced, and the drain was replaced with a 12 Fr silicone drain by using a guidewire on the 15th postoperative day. The tip of the drain was deeply inserted in order for the drain to enter into the cavity (Figs. 2 and 3). At that time, the guidewire insertion operation was difficult, and the guidewire was vigorously inserted. When inserting the drain, there was a high degree of resistance, but the drain was finally inserted. Afterwards, the blood was discharged from the drain, and the drain was closed on the same day. When the drain was opened the next day, blood was again discharged. A contrast-enhanced CT scan showed that the tip of the drain had pierced the inferior vena cava (IVC) from the root of the middle hepatic vein and had become placed into the right RA (Fig. 4). Because 1 month had not passed after the operation, a high degree of adhesion at the surgical site was expected. Therefore, the operation was assessed to be difficult. Additionally, there were risks of intraabdominal hemorrhage and of bleeding into the cavity of the abscess. There was also the risk of a fibrin embolism that may have occurred due to the presence of fibrin. Thus, after consultation with the cardiovascular surgeon about hemostasis, and the risk of performing a thrombectomy, due to the sudden changes, the tip of the drain was removed from the IVC by using fluoroscopy under general anesthesia. At that time, it was confirmed by the use of transesophageal echocardiography that the tip of the drain had been removed to the outside of the area of the IVC. Afterwards, there was no incidence bleeding from the drain, but a persistent fever of 39 °C continued to occur. Echocardiography was performed to investigate the heat source, and a 3.0 × 1.4 cm mass with good mobility was discovered, extending from the IVC to the RA. A contrast-enhanced CT scan demonstrated a series of low density areas, extending from the abscess cavity to the transected liver surface and to the RA. Although removal by the use of interventional radiology was considered, it was decided that there was a risk of a complication of pulmonary embolism due to the large and movable mass, and it was decided that emergency open heart surgery should be performed for the removal of the tumor (Fig. 5). A right atrial incision, thrombus removal, and a middle hepatic vein merging section closure surgery were performed. When the RA incision was performed and the lumen was confirmed, and approximately 5 mm of the perforated portion was located near the root of the middle hepatic vein, and at the same site approximately 3 cm of a yellow-brown mass was discovered. The drain and the mass was removed and the middle hepatic vein was sutured and closed (Fig. 6). The operation time was 142 min, and the intraoperative bleeding was 400 ml. Histologically, the mass was primarily composed of fibrin thrombus, and a collection of neutrophils were primarily observed at the peripheral portion of the mass (Fig. 7). A tissue culture examination detected the presence of *Enterobacter aerogenes*.

The fever was promptly relieved and inflammation and infection gradually improved after the open heart surgery, and the patient was transferred to the hospital for cardiac rehabilitation at 68 days after the first operation.

## Discussion

The rate of bile leakage from the transected surface after a hepatectomy is 3.6% to 10%, which is still one of the most frequent postoperative complications [2–5]. An internal biliary stent is inserted in anastomosis to decrease anastomotic complication [6] and a drain is often placed into the dissected surface of the liver for the early diagnosis of bile leakage [7]. However, if the rate of bile leakage does not improve, or if it is accompanied by both an infection and a drain obstruction, then a drain exchange is required to improve the drainage [8]. At that time, the utilization of a guidewire is generally recommended for guiding the tip of the drain to the site of fluid collection under fluoroscopy. In this case, in order to drain the abscess cavity that was caused by the bile leakage under the right diaphragm, the drain was deeply inserted into the right side, and the middle hepatic vein may have been punctured by the use of the guidewire operation.

The careful performance of the guidewire operation is required for the exchange of the drain. Guidewire operations are performed in a protective manner, and it is recommended that the insertion of the drain is stopped and released slowly at the exact moment before the tip of the guidewire is removed. It is thought that the stiffness of the guidewire is increased when the tip of the guidewire is slightly protruded. Therefore, care must be undertaken, especially when the drain is in vertical contact with the vessel wall. In fact, it had been reported that a straight type of guidewire that is used at the time of central venous catheter placement from the right internal jugular vein can puncture the blood vessel wall of the right brachiocephalic vein located on the extension of the right internal jugular vein [9]. In addition, cases with severe complications such as cardiac tamponade in which the heart is punctured by the use of a guidewire have also been reported [10]. To avoid these complications, J-type guidewires with flexible tips are commonly used, but cases with similar issues, as well as fatal cases, have been reported [11]. In our case, the guidewire that we used was an angle-stiff type of guidewire with a diameter of 0.035 in, and the tip of the drain had a flexibility of 3 cm; however, its stiffness was higher compared to other guidewires, and it was a straight type of guidewire, compared to the J-type guidewires. The root of the middle hepatic vein was located on the extension of the drain before the replacement, and it was thought that the vessel wall was torn during the guidewire operation. It was also possible that the bile leakage and intraabdominal abscess caused the vulnerability of MHV [12].

As a result of searching for the keywords “peritoneal drain” and “straying” in PubMed, no case reports of peritoneal drains that strayed into blood vessels were discovered. Conversely, Chandrashekhara et al. reported a case in which the tip of the percutaneous drain, which was placed for a liver abscess, was misplaced into the IVC [13]. Because it was removed as quickly as possible, it was thought that, without forming a fibrin sheath, no damage had occurred. It was reported that the cause of the tip of the drain misplacement was due to the lack of confirmation of the insertion distance and the site of the tip.

## Conclusion

In the case of drain exchanges that use a guide wires, damage to the surrounding tissues and organs may occur at the tip of the guide wire when the guide wire is inserted deeper than the drain tip. It is necessary to confirm a majority of the enough surrounding tissues and organs with CT scans. In particular, more careful operations are needed when exchange drains are placed near blood vessels.

## Abbreviations

CT: Computed tomography
IVC: Inferior vena cava
RA: Right atrium
